# The Role of Copper Overload in Modulating Neuropsychiatric Symptoms

**DOI:** 10.3390/ijms25126487

**Published:** 2024-06-12

**Authors:** Mirko Manchia, Pasquale Paribello, Martina Pinna, Gavino Faa

**Affiliations:** 1 Unit of Psychiatry, Department of Medical Sciences and Public Health, University of Cagliari, 09124 Cagliari, Italy; pasqualeparibello@gmail.com; 2 Unit of Clinical Psychiatry, University Hospital Agency of Cagliari, 09124 Cagliari, Italy; 3 Department of Pharmacology, Dalhousie University, Halifax, NS B3H 4R2, Canada; 4 Forensic Psychiatry Unit, Sardinia Health Agency, 09123 Cagliari, Italy; martina.pinna@aslcagliari.it; 5 Department of Medical Sciences and Public Health, University of Cagliari, 09124 Cagliari, Italy; gavinofaa@gmail.com; 6 Department of Biology, College of Science and Technology, Temple University, Philadelphia, PA 19122, USA

**Keywords:** genomics, rare diseases, neuropsychiatry, trace metals, toxicity, CNS

## Abstract

Copper is a transition metal essential for growth and development and indispensable for eukaryotic life. This metal is essential to neuronal function: its deficiency, as well as its overload have been associated with multiple neurodegenerative disorders such as Alzheimer’s disease and Wilson’s disease and psychiatric conditions such as schizophrenia, bipolar disorder, and major depressive disorders. Copper plays a fundamental role in the development and function of the human Central Nervous System (CNS), being a cofactor of multiple enzymes that play a key role in physiology during development. In this context, we thought it would be timely to summarize data on alterations in the metabolism of copper at the CNS level that might influence the development of neuropsychiatric symptoms. We present a non-systematic review with the study selection based on the authors’ judgement to offer the reader a perspective on the most significant elements of neuropsychiatric symptoms in Wilson’s disease. We highlight that Wilson’s disease is characterized by marked heterogeneity in clinical presentation among patients with the same mutation. This should motivate more research efforts to disentangle the role of environmental factors in modulating the expression of genetic predisposition to this disorder.

## 1. Introduction

### The Physiological Role of Copper in Physiology in the Central Nervous System

Copper, a transition element crucial for growth and development, is indispensable for eukaryotic life [1]. Its importance extends to neuronal function, with both deficiency and excess linked to various neurodegenerative disorders [2]. Disruption of copper homeostasis may be the basis of the insurgence of ischemic heart disease and neurodegenerative disorders [3]. Only discrete amounts of copper are required physiologically: a delicate balance between copper uptake, copper excretion, and the subcellular distribution of copper ions is fundamental to avoid excess copper and copper deficiency [4]. The reference range of recommended dietary intake varies depending on the considered guideline, country, age, and physiological state (e.g., pregnancy, breastfeeding), but for adults, it typically ranges between 1 and 1.3 mg/day [5]. Table 1 summarizes reference ranges reported in the literature for serum copper. 

Copper plays a fundamental role in the development and function of the human CNS, being a cofactor of multiple enzymes that play a key role in physiology during development. Copper enzymes are crucial in various physiological functions within the CNS, including catecholamine synthesis, safeguarding cellular structures from reactive oxygen species, activating neuropeptides and hormones, and facilitating other vital molecular events [9]. Copper enzymes are oxidoreductases, whose reactions are in a range of relatively high redox potentials. Among them are DBH, involved in catecholamine biosynthesis and in the hydroxylation of dopamine into norepinephrine, superoxide dismutase (SOD), the cytosolic cuproenzyme essential for detoxifying free radicals, Cytochrome C Oxidase (COX), a mitochondrial enzyme involved in oxidative phosphorylation and aerobic respiration, and many others [10]. This evidence highlights how disruption in copper-dependent pathways might determine profound alterations in physiological mechanisms, with relevant somatic and behavioral consequences. This review is poised to present data on the role of alterations in the metabolism of copper at the CNS level that might influence the development of neuropsychiatric symptoms. In Table 2, we summarize some of the most studied cuproenzymes expressed in the CNS. In the next sections, we will provide an overview of these aspects, focusing on the neuropsychiatric consequences of copper overload, including that developing in the context of Wilson’s disease (WD).

## 2. Results

The described search strategy yielded a total of 706 unique records, of which 229 were initially selected based on an initial title and abstract screening. After analyzing the full manuscript of the corresponding publications, 23 papers were included in the qualitative analysis. Further records were identified and included in the following Results Section based on the authors’ personal judgement.

### 2.1. Copper Metabolism

#### 2.1.1. Copper Bioavailability

**Figure 1:**
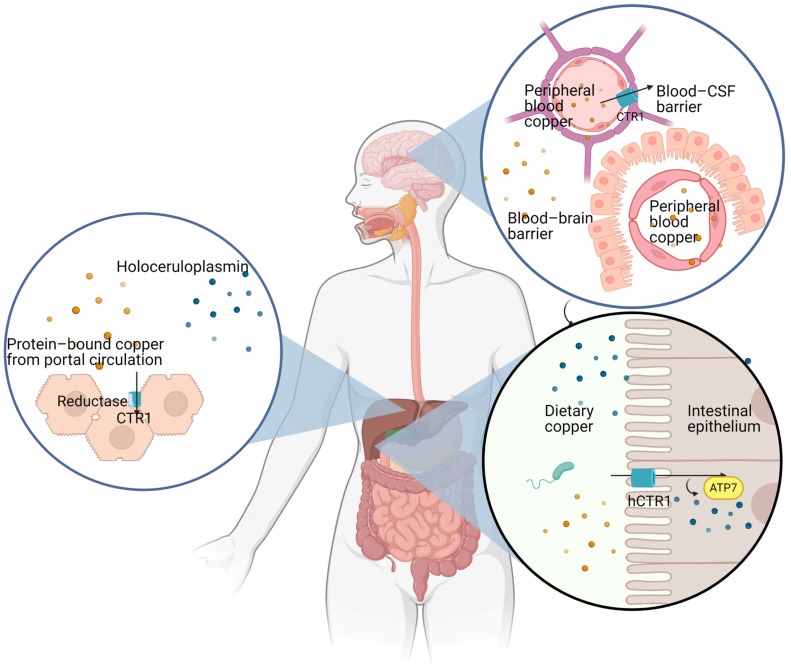
Illustration of the main steps of copper metabolism.

Copper bioavailability depends on the following main factors: (i) copper absorption from the gastrointestinal tract; (ii) copper transport in the portal blood; (iii) copper extraction by hepatocytes from the portal blood; (iv) copper uptake by the CNS through the blood–brain barrier (BBB) and the blood–cerebrospinal fluid barrier (BCB) and by peripheral tissues [16,17] (Figure 1). All these steps, from absorption to transport and uptake of copper ions by different tissues, including the CNS, are regulated by multiple genes encoding for specific copper transporters, which coordinate copper bioavailability [18]. While direct serum copper assessment does not necessarily represent a criterion for suspecting copper overload, this assessment may still have a valuable role in the appropriate clinical scenario for supporting the suspicion of copper deficiency or toxicity related to copper deposition [19]. Several elements may influence copper absorption, including age, gender, type of food, and concomitant medications [5]. The liver’s role in copper handling is crucial in its storage, mobilization, and elimination. Typically, absorbed copper is either excreted through the bile (80%), or a limited amount is released in the blood bound to ceruloplasmin. Therefore, under physiological conditions, copper is not stored in the liver [20]. An exception is represented by the high levels of copper stored in the fetal liver during gestation [21,22]. Excess copper may be shown in tissue sections by multiple histochemical stains, including rhodamine, rubeanic acid [23], and Timm silver stain [24,25]. Attempts to reveal copper overload in tissue sections may produce false negatives: the deparaffination time by xylene is a crucial point in the detection of copper deposits in tissues [26]. 

#### 2.1.2. Copper Uptake and Trafficking in the Brain

The brain cells receive copper from the BBB and through the BCB, with choroid plexus cells playing a key role in regulating copper entry into the CSF and endothelial cells of intraparenchymal capillaries regulating copper entry into the brain parenchyma [27]. In recent years, the neurovascular unit (NVU) has been introduced in the literature as a collective term including all cell types participating in the integrity of the BBB: brain microvascular endothelial cells (BMECs), pericytes, astrocytes, neurons, microglia, and oligodendrocytes [28]. The highest rate of copper ions’ transport into the brain parenchyma, compared to the CSF, clearly indicates that the BBB is the principal site through which copper enters the CNS [29]. Copper transport crossing the BBB and the BCB is regulated by multiple transporters, including the Cu-transporting ATPases ATP7A and ATP7B, both expressed abundantly in the BBB and in the BCFB [30]. In the same study, subcellular trafficking of ATPases was found in choroid epithelial cells: in the presence of copper overload in the choroid plexus cells, ATP7A relocates toward the apical pole of choroid cells facing the CSF, whereas ATP7B relocates toward the basolateral cell membrane facing the blood. Taken together, these data indicate the specific role of ATP7A and ATP7B in the transport of copper ions through the BBB and the BCB, from the blood into the brain or into the CSF and back to the blood [30]. Clearly, ATP7A plays a major role, as compared to ATP7B, in the maintenance of optimal copper levels inside the CNS. The role of ATP7B in copper trafficking in the brain appears more complex and remains partially unknown. It is possible that ATP7B has a prevalent restricted biosynthetic role, participating in copper ions’ incorporation in copper enzymes at the trans-Golgi-network [31]. In immunohistochemical studies focused on the expression of ATP7B in the human brain, this protein was found to be expressed in the cerebellum, substantia nigra, putamen, body of caudate, anterior cingulate cortex, and visual cortex [32]. The highest levels of ATP7B were found in Purkinje neurons in the cerebellum [32]. 

### 2.2. The Influence of Trace Metals Status on Copper Transport in the CNS

Cerebral copper homeostasis may be influenced by other metals, including lead. Pb exposure was found to alter the expression of the membrane copper permease ATP7A, and of the ion channel copper transporter 1 (CTR1) [33,34]. According to these findings, CTR1 and ATP7A play a key role in copper transport in choroidal epithelial cells. Changes in their expression induced by lead exposure may result in copper accumulation in the cerebrospinal fluid and in the brain. More recent studies confirmed the ability of lead exposure to influence the regulation of copper trafficking at the level of the blood–cerebrospinal fluid barrier (BCB), ending with copper accumulation in brain cells [35]. In this study, carried out in rats undergoing lead exposure, the BCB was not able to eliminate the excess copper from the cerebrospinal fluid to the blood [35]. Consequently, copper storage was found in the choroid plexuses, with the dislocation of ATP7A and CTR1 toward the apical pole of the choroid cells. These findings confirmed that lead exposure interferes with copper brain levels, suggesting a possible mechanism of neurotoxicity starting with lead poisoning and ending with copper overload in brain cells. Given that Pb exposure in experimental animals is associated with increased beta-amyloid levels and amyloid plaque formation in the brain [36], Pb-induced copper overload might represent a linkage between lead exposure and the development of Alzheimer’s disease. Moreover, lead exposure has been demonstrated to reduce the clearance of beta-amyloid from the cerebrospinal fluid to the blood by the choroid plexuses, ending with the increase of beta-amyloid in the brain [37].

Other metals, including manganese (Mn), a trace element that is necessary for life, but neurotoxic at high cerebral levels, may change the expression of copper transporters in the blood–cerebrospinal barrier (BCB). Mn exposure induces over-expression of CTR1 in the choroid cells of the BCB, ending with increased copper transport into the brain and copper overload in brain cells [2,33]. The significant role of Mn in maintaining the neuroprotective function of the BBB and the BCB, as well as the molecular mechanisms underlying the regulation of other trace elements trafficking across the BBB and the BCB, have been confirmed in more recent studies [38].

Another transition trace element, iron (Fe), may influence the regulation of brain copper homeostasis, interfering with the brain barrier systems involved in copper uptake from the blood. Iron deficiency may increase the transport of copper ions at the brain barriers, ending with copper overload in the brain cells [39,40]. Although ATP7B is expressed in the brain, copper overload appears to be dependent on liver disease, as it is potentially reversible with a liver transplant [29]. Conversely, in the same study, no significant effect on brain copper homeostasis was observed under iron overload. Iron overload may cause severe hepatic changes [41], leading to hepatic insufficiency with a disarrangement of copper metabolism, ultimately leading to increased copper storage in the brain. Previous studies from our group on trace metal storage in the brain of a patient who was deceased and suffered from WD evidenced the accumulation of copper in multiple brain zones [42]. Specifically, the mean copper concentration in 28 brain samples examined was equal to 125 micrograms/gram of dry tissue versus reference values of 13–60 micrograms/gram of dry tissue [42]. In the same study, we observed a marked decrease in the brain content of multiple elements, including iron and magnesium (20-fold lower than reference values), zinc and calcium (10-times lower than reference values), and phosphorus [42]. Finally, in the brain of the patient having WD, copper storage was uneven, with the highest levels being detected in the peripheral cortical zones [42]. 

### 2.3. Ageing and Copper Stores in Brain Cells

#### 2.3.1. Evidence from Preclinical Models

Intriguing findings regarding the role of ageing on copper transport through the BBB and the BCB have been reported in recent years in experimental models. In rats, the ageing process was associated with relevant subcellular changes in the expression of copper-transporting proteins in the BCB, represented by the choroid plexus. These changes induced an age-dependent decline of copper clearance at the BCB, ending with the tendency of increased copper storage in the cerebrospinal fluid and in the brain [43]. Another study carried out in rats of different ages confirmed an age-dependent increase in copper levels in multiple brain zones. In particular, the subventricular zone (SVZ), a neurogenesis active area bordering the lateral ventricles in strict contact with the cerebrospinal fluid, showed the highest levels of copper storage in old rats, as compared to young and adult animals [44]. Moreover, neuroblasts of the SVZ expressed the highest levels of metallothionines, indicating a key role for these cells in copper detoxification and storage. An interesting study aimed at defining the role of high copper levels in the SVZ using the copper chelator D-penicillamine (D-pen) provided evidence that high copper levels are fundamental for maintaining neurogenesis in SVZ, the neurogenic brain zone in adult animals [45]. During neuronal differentiation, the expression of the plasma copper transporter CTR1 increases, leading to high intracellular copper levels, whereas ATP7A is retained in the trans-Golgi network. In developing neurons, high copper levels are fundamental for ERK1/2 activation, an essential step for the viability of the neurons. In contrast, glial cells show lower copper levels, with ATP7A localized into recycling endosomes in the glial neurites [46]. In North Ronaldsay sheep, characterized by copper overload in the liver and brain, the CTR1 was found to be overexpressed in the BBB and in the BCB choroid cells [47]. This study highlighted the important role of CTR1 in copper uptake into the brain and the cerebrospinal fluid and suggests that aging is associated with dysregulation of CTR1, at the BBB and at the BCB, which might explain the increase of copper levels in the senescent human brain. Cuproptosis is a possible model of the copper-dependent and no-apoptotic mode of regulated death that may represent one of the various mechanisms of altered copper metabolism resulting in synaptic plasticity damage [48]. In a 2023 report, 4-week-old male mice were exposed to copper in their drinking water for 3 months to test the Cu exposure effect on cognitive functions. The study found that Cu exposure worsens learning and memory, leads to copper overload in the brain and urine, causes neuronal degeneration and oxidative damage, and affects synaptic regulatory mechanisms [49]. The exact mechanism and entity through which such phenomenon might influence cognitive decline is still unclear, but they may represent an additional avenue to explore the complexity of neurodegenerative disorders.

#### 2.3.2. Evidence from Clinical Models

Evidence from preclinical models suggests that the elderly might be more vulnerable to copper brain overload. This element should promote screenings of copper status in all patients who are presenting with symptoms of neurodegenerative disorders, including Alzheimer’s disease. According to this hypothesis, multiple studies have been carried out in recent years focused on copper status in patients who are affected by neurodegenerative disorders and in patients having Alzheimer’s disease [50]. In these studies, plasma copper levels and the genetics of ATP7B were investigated [51]. Moreover, copper imbalance detected in subjects with Alzheimer’s disease, often associated with genetic changes in the ATP7B gene, was correlated with the amyloid hypothesis, leading towards a combined genetic and epigenetic etiology of Alzheimer’s disease, in which copper overload in brain cells might play a major role [51]. Certain lines of evidence suggest Cu derangements may be observed in individuals having schizophrenia, with post-mortem analyses indicating tissue accumulation in the CNS, particularly at the caudate nucleus [52]. The role of inflammation in influencing the clinical phenotype for a subgroup of individuals living with mood disorders such as major depressive disorder (MDD) has been hypothesized for a long period of time, leading to the development and conduction of specific clinical trials investigating the effects of interventions tailored to curb inflammation [53]. The evidence in the field is sparse, and at the current stage, no definitive conclusion can be drawn. One possible explanation for such limited evidence of efficacy could be traced back to the underlying heterogeneity of MDD itself. Pertinent to our current understanding of MDD pathophysiology, Cu accumulation can be associated with inflammation, and indeed, it is of vital importance in the metabolism of monoamines [54]. It is possible that a subset of individuals may feature Cu accumulation as one of the many factors contributing to the development and persistence of symptoms, especially among those subjects presenting atypical clinical pictures and limited response to current treatment. As is the case for other psychiatric conditions, despite growing interest in exploring the potential role of trace elements in psychiatric disorders, in the current state of evidence, no clear clinical recommendation can be proposed for their testing and their interpretation for this purpose [54,55].

### 2.4. The Role of Astrocytes in Copper Trafficking

Astrocytes are the first brain parenchymal cells to receive copper ions that cross the BBB, being strategically intermingled between the endothelial cells of the BBB and neurons. As a consequence, astrocytes play a key role in copper transport from the intracerebral capillaries into the brain [10]. hCTR1 and ATP7A are both involved in copper uptake at the astrocytic cell membrane. ATP7A has been postulated to have a role even in the distribution of copper ions from astrocytes to neurons [29]. Interestingly, in an experimental model of copper storage, the excess brain copper was stored as copper-metallothioneins inside the astrocytes, indicating a relevant role for these cells in copper storage in the CNS [47]. Studies on copper content in different human brain areas carried out with laser ablation–inductively coupled plasma–mass spectrometry (LA–ICP–MS) revealed the highest brain copper levels in the hippocampus [56]. The relevant role of astrocytes in copper metabolism in the brain has been confirmed by studies in cultured astrocytes, which efficiently take up copper ions through CTR1 and the divalent metal transporter 1 (DMT1) and export copper with the involvement of ATP7A [57]. These findings indicate a pivotal role for astrocytes in copper distribution in the brain and suggest that impairment of this astrocytic function might be involved in age-related copper overload and neurodegeneration associated with disturbances of brain copper homeostasis [58]. These findings support the notion of astrocytes as key regulators of copper homeostasis by uptaking, storing, and exporting copper and supplying neurons with copper ions. Moreover, astrocytes express the copper transporters CTR1 and CTR2 and ATP7A [59]. More recent studies highlighted the key role played by astrocytes in supporting neuronal metabolism and function, suggesting that the dysfunction of astrocytes might contribute to neuronal demise, ending with neurodegeneration [60]. 

### 2.5. Copper Chaperons in the CNS

At the level of intracerebral capillaries, which constitute the BBB, copper ions might be imported via CTR1 into the cytoplasm of astrocytes. Imported copper ions rapidly bind to copper chaperones, each of which delivers copper ions to specific sites inside the astrocytic cytoplasm. Copper chaperons act as specific shuttles, each of them being able to carry copper ions towards specific sites inside the cell. Furthermore, the human antioxidant protein 1 (hAtox1) facilitates copper transfer to the secretory pathway, delivering copper ions to ATP7A and ATP7B [61]. Recently, a dual role of ATOX1 has emerged: this 68-amino acid peptide acts both as a copper chaperon and as an antioxidant, protecting cells against oxidative stress [62]. Since ATOX1 is a component of the cellular pathways for the survival and protection of neurons against stress, its expression is fundamental in neurons [63]. ATOX1 binds and delivers copper to ATP7B in the trans-Golgi network (or ATP7A in other cells), promoting the synthesis of copper proenzymes. ATOX1 is a copper-dependent transcriptional regulator contributing to cell multiplication. Mice lacking the ATOX1 gene may face perinatal mortality due to abnormal copper homeostasis. Intracellular ATP7A/B regulates copper levels and participates in copper transport between the cell membrane and various intracellular compartments. ATOX1 interacts with ATP7A/B to regulate its activity during copper transport by modulating ATP hydrolysis. In the rat brain, the highest levels of ATOX1 have been reported in the pyramidal neurons of the brain cortex and of the hippocampus, followed by Purkinje cells in the cerebellum [64]. The key role of ATOX1 in brain cells has been underlined by the ability of its inactivation to inhibit the maturation of cuproenzymes, as well as of copper export from brain cells [65]. Copper binds to ATOX1 to enter the nucleus and regulate signal transduction pathways. ATOX1 interacts with cysteine-rich protein-2 (CRIP2), transferring copper and inducing CRIP2 degradation, increasing reactive oxygen species (ROS) levels, and activating autophagy. Copper also regulates gene expression and protein synthesis by influencing transcription factors, including NF-κB, and activates key transcription factors such as AP-1 and p53 [66]. Taken together, these findings suggest that ATOX1 might be localized at the intersection of different cellular networks that regulate copper distribution inside brain cells, with a key role in the regulation of cellular redox balance. Moreover, ATOX1 has been indicated as a transcription factor, involved in cell proliferation [67].

Finally, Cox17 is a copper chaperone essential for the assembly of COX. COX consists of 11 subunits, requiring 18 proteins for its assembly. Cox17 transports two copper ions to Cytochrome C Oxidase assembly protein 2 (SCO2), which in turn delivers the copper ions to the Cytochrome C Oxidase assembly protein 1 (SCO1) in the SCO1–SCO2 complex, resulting in the formation of the COX2 assembly. Mutations in the SCO1 and SCO2 genes can greatly impact this pathway’s integrity [66]. Moreover, Cox17 is involved in the delivery of copper ions to mitochondria [68]. Cox17 is generally considered a key factor in the maintenance of intracellular copper homeostasis, shuttling copper ions to Sco1/2 for subsequent incorporation into Cytochrome C Oxidase [69].

In summary, copper trafficking inside astrocytes is characterized by the following main steps:

- Copper ions are uptaken at the cell membrane by copper transporter 1 (Ctr1) and divalent metal transporter 1 (DMT1). Given that both Ctr1 and DMT1 can uptake Cu+, a cupric reductase provides the reduction of Cu^2+^ to Cu^+^.
- Excess intracellular copper is stored as complexes with metallothioneins [70].
- Copper ions are shuttled to specific intracellular targets by specific chaperons: CCS delivers copper to SOD1; Cox17 delivers copper to Sco1/2 for subsequent incorporation into Cytochrome C Oxidase inside mitochondria; ATOX1 transports copper ions to ATP7A in the trans-Golgi network for subsequent incorporation into multiple copper enzymes, including ceruloplasmin. When copper stores exceed physiological levels, ATP7A may translocate towards the cell membrane, where it has another function: to export excess copper into the extracellular space or into neurons [59].

### 2.6. Copper in Wilson’s Disease

WD is a genetic disorder of copper metabolism caused by mutations in the ATP7B gene [71,72]. WD is an autosomal recessive disorder of copper metabolism, resulting from the defective activity of a copper-transporting ATPase, ATP7B, localized to the trans-Golgi network of multiple cell types, including hepatocytes and some neurons [73]. The major function of ATP7B is to transport copper into the secretory pathway, having a primary function in the incorporation of copper ions into apo ceruloplasmin, originating ceruloplasmin [74]. When intracellular copper levels are elevated, ATP7B may translocate from the Golgi apparatus to the cell membrane at the biliary pole of hepatocytes, participating in the excretion of excess copper into the bile [75]. This copper-level-dependent dislocation of ATP7B represents an interesting post-translational mechanism of this peculiar ATPase, allowing a dual role of ATP7B: copper incorporation into cupro-enzymes, including ceruloplasmin, and cell protection from the toxic effects of copper overload in conditions of copper stress.

#### 2.6.1. The Multiple Clinical Phenotypes of Wilson’s Disease

WD is a rare disease, with an incidence below 1/10,000 live births worldwide [76], with the exception of some specific populations, including the inhabitants of the island of Sardinia, in which WD shows a higher incidence of approximately 1/3000 live births [77]. patients having WD show a marked interindividual variability regarding genetics, clinical presentation, and progression of the hepatic, neurological, and psychiatric symptoms [78]. Here, we describe some of the phenotypic presentations of WD:

1. Early-onset: WD may present very early in life, often during the first five years of life [79]. Clinical presentation in people who are young is often hepatic, with hepatomegaly. Liver biopsy may evidence liver steatosis, associated with Mallory–Denk bodies, liver cell necrosis, and less frequently, lymphocytic infiltrates in portal tracts [72]. At the ultrastructural level, transmission electron microscopy reveals the presence of marked changes in mitochondria, which show paracrystalline inclusions, detachment of the two membranes, and cristae enlargement [80]. Liver disease may progress, leading to cirrhosis and requiring liver transplant.
2. Late-onset: In some patients, WD may present in adulthood in the absence of a clinical history suggestive of the disease in early life [81]. In rare cases, late onset is associated with a fulminant evolution, which in the absence of a liver transplant, may lead to death [82].
3. Hepatic presentation: Liver disease is considered the typical presentation of WD. It is determined by copper accumulation in the periportal hepatocytes, leading to cell death, including apoptosis and cell necrosis. The histological picture of liver disease may overlap with alcoholic liver disease (ALD), being characterized by steatosis, Mallory–Denk bodies, progressing to liver fibrosis, and ending with the insurgence of liver cirrhosis [71]. Liver involvement may be the predominant clinical presentation, or it may occur in association with neurological and psychiatric symptoms.
4. Neurological presentation [83,84]: There is a high proportion of patients affected by WD who show neurological symptoms, ranging from 40% up to 50% [85]. This epidemiological figure reveals an important role of ATP7B in copper homeostasis in the brain. The mechanisms underlying CNS involvement in WD have not been completely clarified. The brain areas that are more damaged in individuals who are WD carriers are the thalamus, subthalamic nuclei, cerebellum, and frontal cortex [85]. The absence or the malfunctioning of mutated ATP7B might cause the malfunctioning of the cupro-enzyme DBH, expressed in noradrenergic neurons, which catalyzes the conversion of dopamine into norepinephrine. The impaired DBH synthesis and function might explain the abnormalities of basal ganglia that result in the development of Parkinsonian symptoms, including tremors and rigidity [86]. The spectrum of neurological symptoms in patients having WD varies widely and includes dystonia, dysphagia, ataxia, choreoathetosis, rigidity, tremors, Parkinsonism, dysarthria, and coordination defects [87].
5. Psychiatric presentation. The presence of psychiatric disorders in less than 50% of patients having WD indicates that other cofactors, including environmental determinants, should be considered when evaluating the clinical phenotype of patients having WD [88,89]. The spectrum of psychiatric symptoms is wide, including personality change, antisocial behavior, aggressive behavior, sleeplessness, depression, anxiety, altered working performances, and psychotic symptoms, including hallucinations [90,91]. Anecdotally, Wilson’s disease has also been reported to present with a catatonic episode following an acute psychotic episode, with possible negative implications in terms of late diagnosis and treatment initiation [92].
6. Fulminant onset: This dramatic presentation of WD is related to an abrupt release of high amounts of non-ceruloplasmin-bound free copper from the liver into the blood, which causes hemolysis and severe acute anemia and may lead to oxidative stress in multiple organs, ending with multi-organ failure. Plasmapheresis may be an important tool to allow the survival of patients who are affected waiting for liver transplants [93].

#### 2.6.2. Genetics of WD: The Molecular Pathways Involved in Systemic Copper Overload

The genetic basis of WD is complex: a recent study defined WD as “a genetic puzzle with diagnostic implications” [94]. Indeed, more than 900 variants of the ATP7B gene are reported. Due to the high number of mutations, the usefulness of genetic tests in clinical practice remains negligible, so even the most advanced genetic analyses, including new-generation sequencing, require additional biochemical and occasionally histological evidence, including the evaluation of urinary copper excretion and needle liver biopsy to reach a certain diagnosis of WD. In a recent study on the characterization of the salivary proteome of WD, a distinct pattern reflecting oxidative stress and inflammatory condition was associated with the phenotype, indicating that the salivary test is a useful tool for diagnostic purposes [95]. The complexity of the clinical evolution of WD is complicated by the multiple epigenetic factors that may increase the risk of the disease, causing an acceleration of copper overload and toxicity or, alternatively, as in the case of a diet rich in zinc, protecting patients from copper toxicity [96,97]. Mutational analyses carried out in 700 Wilson’s disease families from Mediterranean countries, including Sardinia, allowed the identification of 175 mutations, with the presence of many rare mutations. In the majority of cases, mutations reside in 12 exons of the ATP7B gene, suggesting that WD in the populations that are Mediterranean might result from a limited number of mutations, as compared to the population that is Italian, in which a much higher allelic heterogeneity is present [98,99,100,101,102]. Genetic analyses of patients having WD of Sardinian origin evidenced the six most common mutations accounting for 85% of patients, suggesting the presence of a founder effect [77,100]. Moreover, in the Sardinian population, a 3.8% carrier frequency was found, suggesting a previously unreported high frequency of heterozygotes for WD among Sardinians [77]. In a 2022 paper from China, researchers provided clinical and genetic characterization of variations of the ATP7B gene in subjects with Wilson’s disease [103]. Out of 941 recruited subjects, 665 showed significant neurological symptoms, with dysarthria being independently linked to sex, young age at onset, and the A874V mutation. Moreover, the PTV, R778L, and P992L mutations were more commonly found in patients having an early onset, while the A874V mutation was more prevalent in patients having a late onset. Patients having the R778L/A874V genotype had a later onset age compared to those having either the R778L/R778L or R778L/P992L genotypes [103]. 

#### 2.6.3. Psychiatric Symptoms in WD

There is consistent evidence showing that severe psychiatric disorders occur frequently in WD. For instance, the analysis of a multisite international WD registry showed that about thirty-seven percent of patients had a lifetime history of major depressive disorder, while 6% met the criteria for an active major depressive episode [104]. There are anecdotal reports of mania secondary to WD [105], and it is of interest that electroconvulsive therapy appears to be effective [106]. A recent review suggested that 30% to 40% of patients show psychiatric manifestations at the time of diagnosis of WD, and 20% had seen a psychiatrist prior to their WD diagnosis [88]. Interestingly, the latency between the psychiatric symptoms and the diagnosis of WD was quantified at 864.3 days [88]. Finally, there is wide heterogeneity in the prevalence of psychiatric disorders in patients having WD, ranging from 4 to 47% for major depressive disorder and 1.4 to 11.3% for psychosis [88]. Interestingly, it has also been shown that some mutations in the ATP7B gene could be associated with specific psychopathological symptoms and personality traits [107]. For instance, patients who are homozygous for the Trp779Stop and the Thr977Met mutations had high scores on Psychopathy-related scales, whereas patients having His1069Gln/Arg1319Stop mutations had the lowest scores on these scales [107]. However, the evidence for the association of specific ATP7B gene variations with a specific pattern of psychopathology is, at best, preliminary. The same genetic variation may be associated with a highly varied clinical course, with scarce evidence linking specific genotypes and the high phenotype variability [108]. Anecdotally, a case report in 2020 poignantly described that, among sisters carrying the same genotype for the ATB7B gene [c.3207C > A/c.3904-2A > G], one presented prominent neuropsychiatric symptoms (i.e., tremors, motor incoordination, language and cognitive impairment), whilst the other featured solely liver involvement [109]. These extreme examples are far from being interesting trivia, but rather further underscore the complexity of interpreting at large the available evidence in the field and the importance of epigenetic factors in influencing the phenotype, even for monogenic disorders such as WD. Similarly, the evidence for a correlation between peripheral copper concentrations and neuropsychiatric symptoms, albeit promising, is far from being definitive or clinically viable.

### 2.7. Possible Psychiatric Symptoms Related to Copper Derangements

Certain lines of evidence suggest that exposure to a mixture of environmental metals during adolescence (i.e., copper, manganese, zinc, lead, chromium) may be associated with abnormalities in the information integration at the brain network levels locally and on a global level on resting-state functional Magnetic Resonance Imaging (fMRI) [110]. Copper blood concentration anomalies have been increasingly reported in association with Autism Spectrum Disorders (ASDs), with aberrant nutritional and metabolic copper profiles being a possible biomarker candidate for a subset of individuals having ASDs [111]. Attention-Deficit Hyperactive Disorder (ADHD) has also been investigated in the context of establishing the potential worth of copper peripheral blood levels as a possible clinically viable biomarker. Despite the relatively limited sample size currently assessed, preliminary reports suggest a tendency for higher copper levels among ADHD children as compared with controls, with a possible association of the copper/zinc ratio and teacher-rated score for inattention [112]. A further report described a significant association between serum copper and sleep duration, leading to speculations surrounding a possible role of copper itself in influencing sleep regulation among individuals having schizophrenia (SCZ) [113]. A study investigating the possible role of nutritional biomarkers in SCZ found high serum B12 and a high free red blood cell copper-to-zinc ratio significantly associated with symptom severity, despite these findings failing to substantiate a possible clinically viable role for this biomarker panel for diagnostic purposes [114]. Interestingly, past reports investigated the possible association of copper peripheral concentrations and treatment-resistant depression, concluding that no clear association could be established between total severity score (Montgomery–Asberg Depression Rating Scale (MADRS) and Young Mania Rating Scale (YMRS)), but that serum copper concentrations appeared significantly higher before starting ketamine infusion treatment [115]. Data from the National Health and Nutrition Examination Survey further suggest a possible role for copper metabolism derangement in the development of depressive symptoms. From a total of 4552 individuals who are adult, Huang et al. reported a significant association between higher serum copper concentrations and Patient Health Questionnaire-9 (PHQ-9)-defined depressive symptoms [116]. Higher peripheral copper has also been reported to be associated with a higher frequency of mild cognitive impairment, with a stronger association among individuals who are elderly and male [117]. Based on the exploratory nature of the reported data, as mentioned in previous sections of this manuscript, no firm conclusion can be drawn either on the worth of the association of copper and the studied clinical outcomes or on their clinical viability.

## 3. Discussion

In recent years, there have been relevant developments in the comprehension of the molecular pathways involved in the maintenance of copper homeostasis in the CNS, which currently appears as an ensemble of multiple proteins integrated into a regulatory copper network. The recognition of the dysregulation of copper homeostasis as a key pathological feature not restricted to rare diseases due to inborn errors of copper metabolism (WD [80], Menke’s disease [118], Mednik syndrome [18]), but also present in neurodegenerative disorders such as Parkinson’s disease and Alzheimer’s disease [50,51], as well as in prion disease [119,120] has induced several researchers to focus on the study of the molecular pathways involved in the control of copper homeostasis in health and in disease. In the complex network regulating copper trafficking among cells and inside the cytoplasm, ATP7A and ATP7B appear as its most critical components. Both proteins catalyze copper transport across cell membranes, have a key role in the removal of excess copper from the cell, and are deputed to the metalation of multiple cuproenzymes fundamental for cellular life when located in the trans-Golgi network. Their deficiency due to gene mutations is at the basis of WD (mutations of the ATP7B gene) and Menke’s disease (mutated ATP7A gene), two disorders characterized by severe neuropathological deficits and by neuropsychiatric symptoms in a high percentage of patients who are affected. Clearly, the fundamental roles played by copper ions in neuronal and astrocytic function in physiology and the complex consequences in these cells following dysfunction of the copper network, resulting in copper overload or copper deficiency, represent the explanation for the complexity of neurological and psychiatric symptoms that characterize clinical presentation of the patients who are affected. On the other hand, the complexity of copper metabolism is at the basis of the heterogenous clinical presentations of WD, as well as Alzheimer’s disease, both characterized by copper dysregulation. Intriguingly, WD shows marked differences in clinical presentation even among patients who are carrying the same mutation. Some patients have a very early onset, with liver disorders starting at five years of life; others have a later onset, in adulthood, and a small proportion of rare cases start at 70 years. Some patients have predominant liver involvement, leading to the insurgence of juvenile cirrhosis; others show a neurological involvement in association with psychiatric symptoms; others have all types of manifestations. It is reasonable to state the following: (i) genetics is not able, alone, to explain the severity of the disarrangement of copper metabolism; (ii) epigenetics may play a relevant role in the clinical phenotype [97], starting with the maternal diet during gestation, which when not well controlled to avoid copper overload in the developing brain and liver, may have detrimental effects on neurons; (iii) the isolated mutation of ATP7B may have detrimental effects on copper trafficking, but the association with one or more mutations in the gene of another copper chaperone involved in the metabolism of this metal may cause a more heterogeneous and severe clinical manifestation. The combined impact of ATP7B genetic mutations and epigenetic changes on the phenotype remains unexplored. In summary, Wilson’s disease (WD) presents a genetically complex phenotype regulated by molecular genetics and epigenetic mechanisms [96].

## 4. Methods

The present manuscript aims to integrate and synergize the complementary expertise of study authors to yield an updated perspective on the known role of copper overload in neuropsychiatric disorders and on the potential prospects for this area of research. We performed a non-systematic review with two authors (P.P. and M.M.), selecting relevant papers based on personal judgment and predetermined criteria. To enrich our study selection, we applied the following search strategy on Pubmed, ((((mental health) OR (psychiatry)) OR (neuropsychiatry)) OR (neuropsychiatric)) AND (copper), and focused only on papers published in the last 10 years (i.e., from January 2014 onwards). G.F. and M.P. focused on studies involving the pathophysiology mechanism of disorders and further oversaw the drafting of this manuscript. Additionally, a comprehensive pearl-growing strategy was applied to broaden the scope of the research project, including pertinent reviews, books, and other sources. The study content is summarized and presented in brief paragraphs. Finally, a detailed discussion highlights the most significant findings from the selected papers.

## 5. Conclusions

An accurate epidemiological estimate for neuropsychiatric symptoms related to copper overload appears plagued by underreporting and underdiagnosis, as is often the case for other conditions reported to be associated with psychiatric disorders. The present review offers a summary of some of the existing literature in the field, underscoring existing knowledge gaps and, specifically, in terms of the still largely unknown role of the interaction between the environment and pre-existing risk profiles in producing atypical neuropsychiatric presentations (e.g., epigenetic changes).

## Figures and Tables

**Table 1:** Reference ranges for serum copper levels according to age.

Age Range	Reported Concentration (µmol/L)
Children < 10 y.o.	11.8–24.0 [6]
Children 10.2–12.5 y.o.	8.9–20.3 [6]
Children > 12.5 y.o.	8.9–20.3 [6]
Men 20–50 y.o.	14.2–17.6 [7].
Post-menopausal women	15.1–19.5 [8]

Abbreviations: y.o.—years old; µmol/L—Micromoles per Liter.

**Table 2:** Summary of some of the most studied enzymes requiring copper with possible implications for CNS disorders.

Involved Enzymes	Known Physiological Functions	CNS Disorder
Dopamine beta-hydroxylase	Catalyzes the formation of norepinephrine from dopamine	Neurodegenerative (Alzheimer’s and Parkinson’s disease), neuropsychiatric (schizophrenia, major depressive disorder) [11]
Lysyl oxidase	Promotes extracellular crosslinking of collagen and elastin	Alzheimer’s disease [12]
Tyrosinase	Key enzyme in melanin synthesis	Parkinson’s disease [13]
Superoxide dismutase 3	Antioxidant enzyme	Amyotrophic Lateral Sclerosis, Huntington’s disease, Parkinson’s disease, Alzheimer’s disease [14].
Cytochrome C Oxidase	Cellular energy	Alzheimer’s disease [15].

## Data Availability

Not applicable.

## Abbreviations

ALS, Amyotrophic Lateral Sclerosis; ALD, alcoholic liver disease; ATP7A, ATPase Copper Transporting; Alpha ATP7B, ATPase Copper Transporting Beta; BBB, blood–brain barrier; BCB—blood–cerebrospinal fluid barrier; COX, Cytochrome C Oxidase; CTR1, copper transporter 1; Cu, copper; CSF, cerebrospinal fluid; DBH, dopamine beta-hydroxylase; DMT1, divalent metal transporter 1; ERK1/2, Extracellular Signal-Regulated Kinase 1/2; Fe, iron; hAtox1, human antioxidant protein 1; hCTR1, human copper transporter 1; LA–ICP–MS, laser ablation–inductively coupled plasma–mass spectrometry; Mn, manganese; NVU, neurovascular unit; Sco1/2, Synthesis of Cytochrome C Oxidase 1/2; SOD, superoxide dismutase; SOD1, superoxide dismutase 1; SVZ, subventricular zone; WD, Wilson’s disease; y.o., years old; µmol/L, Micromoles per Liter
